# “Death audit is a fight” – provider perspectives on the ethics of the Maternal and Perinatal Death Surveillance and Response (MPDSR) system in Ethiopia

**DOI:** 10.1186/s12913-022-08568-0

**Published:** 2022-09-29

**Authors:** Kaya Cetin, Dawit Worku, Asrat Demtse, Andrea Melberg, Ingrid Miljeteig

**Affiliations:** 1 Bergen Center for Ethics and Priority Setting, University of Bergen, Bergen, Norway; 2 Addis Center for Ethics and Priority Setting, Addis Ababa University, Addis Ababa, Ethiopia

**Keywords:** Death audit, Ethiopia, Perinatal health, Medical ethics

## Abstract

**Background:**

Maternal and neonatal health are regarded as important indicators of health in most countries. Death auditing through, for example, the Maternal and Perinatal Death Surveillance and Response (MPDSR) is viewed as key to preventing maternal and newborn mortality. However, little is known about the implications of implementing perinatal auditing for healthcare professionals in low-income contexts. This study aimed to explore the ethical and practical consequences clinicians experience concerning MPDSR reporting practices in Ethiopia.

**Methods:**

Qualitative semi-structured in-depth individual interviews were conducted with 16 healthcare workers across professions at selected facilities in Ethiopia. The interview questions were related to clinicians’ experiences with, and perceptions of, death auditing. Their strategies for coping with newborn losses and the related reporting practices were also explored. The material was analyzed following systematic text condensation, and the NVivo11 software was used for organizing and coding the data material.

**Results:**

Participants experienced fear of punishment and blame in relation to the perinatal death auditing process. They found that auditing did not contribute to reducing perinatal deaths and that their motivation to stick to the obligation was negatively affected by this. Performing audits without available resources to provide optimal care or support in the current system was perceived as unfair. Some hid information or misreported information in order to avoid accusations of misconduct when they felt they were not to blame for the baby’s death. Coping strategies such as engaging in exceedingly larger work efforts, overtreating patients, or avoiding complicated medical cases were described.

**Conclusions:**

Experiencing perinatal death and death reporting constitutes a double burden for the involved healthcare workers. The preventability of perinatal death is perceived as context-dependent, and both clinicians and the healthcare system would benefit from a safe and blame-free reporting environment. To support these healthcare workers in a challenging clinical reality, guidelines and action plans that are specific to the Ethiopian context are needed.

## Background

Mortality rates of mothers and newborns are considered important indicators of health and development in all countries [1], and the prevention of maternal, newborn, and child deaths is part of the Sustainable Development Goals declared by the United Nations [2]. Ethiopia, a rapidly developing country with a relatively young population, has in recent years made great efforts to reduce the national perinatal mortality rate [3].

The latest numbers indicate that yearly 2.4 million newborns die and nearly 2 million are stillborn globally [4, 5]. Sub-Saharan Africa carries the burden of 44% of global stillbirth and neonatal deaths. Although the focus on reproductive, maternal, neonatal, and child health interventions has been reinforced in Ethiopia, the mortality of newborns is not decreasing as expected when considering reductions in mortality rates among older children and mothers [6, 7]. Persistently, the most common causes of neonatal death are preterm birth complications, birth asphyxia, and sepsis [8, 9].

**Fig. 1:**
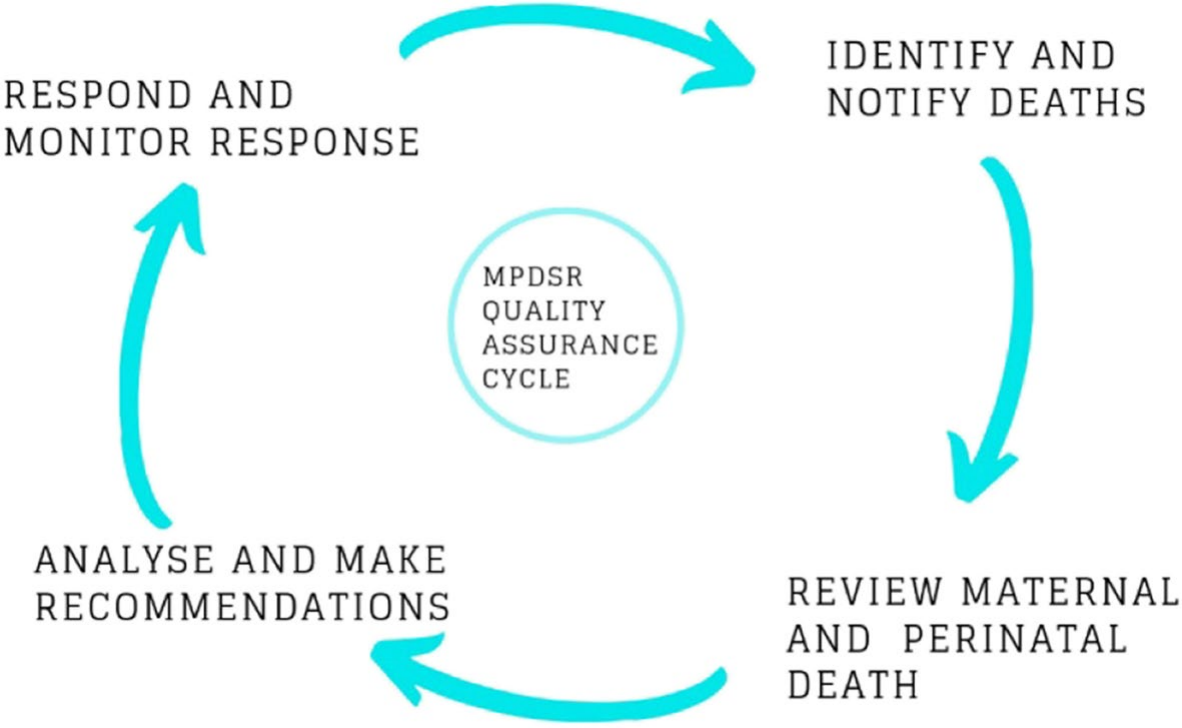
The MPDSR cycle adapted from WHO [12]

**Fig. 2:**
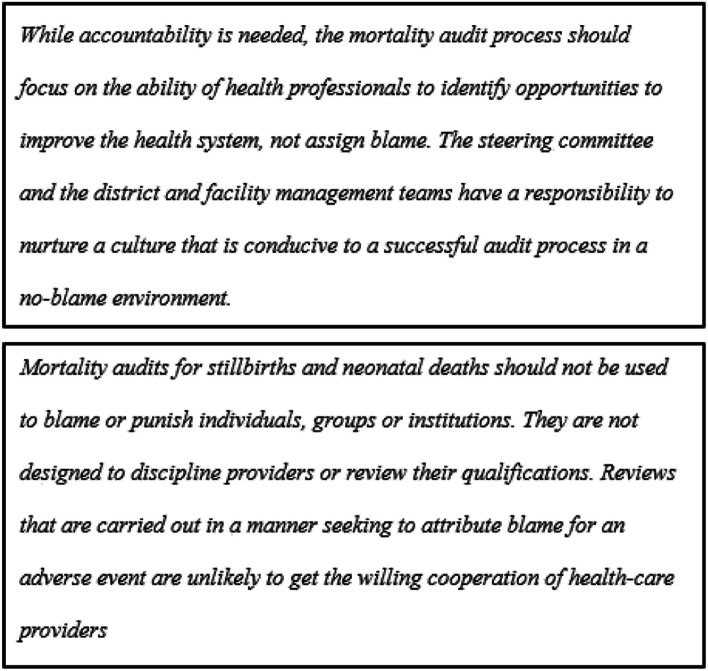
Extract from the WHO guidelines on the MPDSR, advocating a no-blame policy [12]

While there is a general agreement that registration and surveillance of newborn deaths are needed to better understand the causes of perinatal deaths and to drive actions for mortality reduction, the reporting practices and their implications for healthcare providers on the facility level are not well known. The Maternal and Perinatal Death Surveillance and Response (MPDSR) system was introduced by the World Health Organization (WHO) as a tool for understanding the causes of maternal and perinatal death. Understanding these causes should in turn drive actions for reducing mortality and increasing the quality of healthcare worldwide [10, 11]. The system follows a step-by-step action cycle and advocates a no-blame policy, see Figs. 1 and 2. MPDSR implementation has received significant political commitment in Ethiopia. The tool is addressed in the Health Sector Transformation Plan, and data from the MPDSR are referred to and used as the basis for healthcare priority setting in the Essential Health Services Package of Ethiopia [3, 6].

Barriers to implementation and the process of maternal death review have been subject to some research in Sub-Saharan African countries, including Ethiopia [10, 13–18]. The process of auditing and reviewing perinatal deaths has, however, received less attention. To our knowledge, no assessment of perinatal reporting practice and healthcare workers’ resilience has been done in Ethiopia.

Navigating ethically challenging situations is part of the job for healthcare providers everywhere, and moral distress occurs when deep-rooted values or beliefs are being contested without the possibility of amendment [19, 20]. Moral distress is well explored among healthcare professionals in high-income settings, ranging from medical doctors to healthcare students [19, 21], but in the context of sub-Saharan Africa moral distress is not widely described [22–26]. Experiencing ethically challenging situations without proper support or strategies to handle them may lead to moral distress, and this may in turn lead to physical and psychological reactions and often leads to conflicts, burnout, and personnel leaving their jobs [27, 28]. Moral resilience has been described in clinicians as the individual’s capacity for responding constructively to ethically challenging situations of moral distress [29], and ways in which to cultivate moral resilience include “fostering self-awareness” and “speaking up with clarity and confidence”, among others.

We argue that acquiring more knowledge on experienced ethical challenges and moral distress associated with perinatal death reporting practices can be a valuable input in strategies to train, support, and motivate healthcare providers as well as for ensuring that the policies and guidelines are suited for the clinical reality. In this paper, we aimed to explore the experiences and challenges clinicians face in relation to neonatal death reporting practices in Ethiopia. Specifically, we sought after i) what kind of perinatal death reporting practices are in place, ii) how are perinatal death reporting practices perceived by healthcare providers, and iii) how healthcare providers manage the related challenges they encounter.

## Methods

Clinicians’ perceptions of death auditing, in addition to their strategies for coping with newborn losses, were sought after in this qualitative study. Semi-structured in-depth interviews were conducted by the first author and second author from February to March 2020.

### Study setting and participants

Ethiopia was in 2013 among the first countries in Africa to implement the Maternal Death Surveillance and Response (MDSR) system. From 2016, perinatal deaths were also included in this initiative, extending to the MPDSR. Table 1 provides some sociodemographic characteristics of the nation. The obstetrics/gynecology departments and neonatal/pediatrics departments at government hospitals in urban cities were selected through purposive sampling. To give an overview of healthcare services given to mothers and neonates, study sites with a high patient flow and likely high occurrence of perinatal deaths were sampled. Large public tertiary referral hospitals receive a high patient burden and a high rate of complicated deliveries with a higher likelihood of negative birth outcomes. The implementation of MPDSR receives significant attention in the public health sector.

**Table 1:** Sociodemographic indicators of Ethiopia [4, 5, 30–34]

Population indicators	
Total population (1000 s)	112,079
Life expectancy at birth (years)	68.7
Total fertility rate (births per woman)	4.3
Maternal mortality rate (deaths per 100,000 live births)	401
Neonatal mortality rate (per 1000 live births)	26.9
Estimated stillbirth rate (per 1000 total births)	24.6
Under-five mortality rate (per 1000 live births)	48.7
Physicians per 10,000 population	0.8
Population living below the income poverty line of $1.90 /day (%)	30.8
Health expenditure per capita (current US $)	24.2

To obtain nuanced data on the topic, participants were purposefully recruited with a focus on various roles and professions in the clinic and regular involvement in maternal and perinatal healthcare [35]. To gain insight from routine chores in relation to perinatal health, participants were recruited while attending daily work at the chosen clinics.

### Data collection

Interviews with 16 healthcare workers were conducted. The interviews consisted of open-ended questions such as “What is most challenging about being a [doctor/nurse] here” and probing questions such as “Can you tell me more about the audit process “. The interview guide was revised after pilot interviews and continuously adapted throughout the fieldwork. Recruitment of participants ended when no major new themes emerged in the analysis, i.e. when approaching thematic saturation [36]. Interviews lasted approximately 45 to 60 min and were digitally recorded and transcribed.

### Data analysis

Transcriptions of interviews were analyzed continuously as the fieldwork proceeded following Malterud’s systematic text condensation analysis [37, 38]. We started by gaining an overall understanding of the material, identifying the units of meaning, and coding them. Within the coded groups, we extracted meaning and condensed each coded group into summarized descriptions of the most important triggers, reactions, and opinions. NVivo11 software was used for organizing and coding the data material.

### Ethics approval

The study was approved by the Regional Committee for Medical and Health Research Ethics, Western-Norway (REK Vest, project 7103), Addis Ababa University College of Health Sciences Institutional Review Board (AAU IRB, FS No 00019188) and Norwegian Centre for Research Data (reference code 494,369). Potential participants were informed about the objective of the study, that anonymity would be ensured, that participation was voluntary, and that consent could be withdrawn at any given time during the study period before publication. Written informed consent was obtained by all participants before the interviews. Neonatal death and its reporting practices is a sensitive issue for the involved clinicians, and this was recognized and taken into account during interviews. To ensure anonymity, details on our study sites are left out.

## Results

All participants had experienced perinatal deaths and death audits in their work in the neonatal or obstetric units. In the following section, we present how perinatal audits were practiced at the study sites. We also present healthcare professionals’ perceptions of perinatal audits and their various coping strategies when faced with the loss of patients, chaotic emergency situations, and the obligation to report and attend audit meetings. Respondent characteristics are summed up in Table 2.

**Table 2:** Demographic overview of the participants in the study

		Number of participants (n)
Gender	Women	6
	Men	10
Age group	20–25	2
	26–31	8
	32–37	4
	38–44	1
	45–50	1
Professional status	Midwife	3
	Nurse (pediatrics)	1
	Senior doctor^a^	5
	Junior doctor^b^	6
	Intern	1
Years in practice	0 to 4	5
	5 to 10	9
	11 +	2

^a^Senior doctor refers to doctors who have completed their years of residency and are practicing medicine as specialists in gynecology, obstetrics, or pediatrics

^b^Junior doctor refers to doctors who are currently undertaking their residency in gynecology, obstetrics, or pediatrics, and who have yet to be considered qualified specialists within these fields of medicine

### The practices of perinatal death reviews

Perinatal audit meetings were carried out differently at the study sites. While the frequency of meetings and number and character of the invited participants varied, the content was largely similar. Some departments had weekly audits, reviewing all cases of perinatal deaths from the past week, while others conducted monthly audit meetings with a larger audience, presenting a few selected cases from the previous month. For example, one department held audit meetings towards the end of every month, on two separate subsequent days. All staff involved in perinatal healthcare and hospital managerial staff were invited. The meeting was considered mandatory for medical interns and junior doctors, while others, such as managerial staff, were invited but not obligated to attend. The total number of attendees could vary from 15 to 35. An appointed doctor in his/her last year of residency, referred to as a junior doctor, was responsible for presenting data from patient charts documented on paper and for gathering statistics from the wards. Statistics including the number of deliveries, mode of delivery, gestational age, and mothers’ parity in addition to perinatal mortality rates were presented. In addition, a few [1–3] de-identified cases of preventable perinatal loss were selected by the appointed junior doctor for specific case presentation and discussion. After the presentations, senior doctors typically inquired about the management of the cases. The junior doctor who presented was expected to answer these questions, and the rest of the audit attendees would join the discussion. Other junior doctors and interns were present but silent. The cases presented and discussed in the audit meetings were chosen by the appointed junior doctor and were selected based on the criterion of preventability.

### Preventable deaths only

Although cases were only eligible for presentation if the perinatal death was considered preventable, the participants reported that the guidelines did not specify what avoidable or preventable deaths entailed. Participants voiced reflections on how access to resources influenced this matter, and what is considered avoidable in one setting may very well be seen as an expected and unpreventable neonatal death in a setting with fewer resources. Exemplifying the latter, participants pointed out that auditing the death of neonates with a gestational age of 28 weeks or a birth weight of 1.2 kg would be unthinkable even though these neonates would be treated and likely survive in a setting with higher access to resources:

*[...] They are avoidable, I think. But the issue is we just… I don’t know. Maybe it is lack of manpower or lack of resources and to some degree, we are accepting more patients than we can handle. So that is a very significant issue. We do the best that we can with the resources at hand, but that doesn’t mean that it is the best practice with the best results, you know. It just doesn’t guarantee anything.* (Junior doctor)

### Duty, burden, and blame—perceptions of perinatal death audits

Perinatal audit meetings were regarded as an important duty for educational purposes. Several pointed out the need to learn from mistakes and the critical situations to improve practice and prevent future mistakes.

*You know, in these audits, we don’t want to accuse the nurse who committed that, the interns who committed them, the resident or the senior. But we use them to learn for the future.* (Junior doctor)

However, direct questioning, naming, and identification of the involved clinicians did occur during case audits, and they were therefore not considered a purely academic exercise by our participants.

*The most difficult thing at the internal audit is the blaming issue, in audit by nature. Usually, the team that is involved feels that they are blamed, but because usually the discussion involves, like, ‘’what happened?’’ Because you go step by step. It is natural that the responsible health professionals will be identified.* (Senior doctor)

Especially younger doctors and nursing staff found senior doctors and other staff to focus chiefly on negligence and on placing blame on the responsible individuals.

*We, the nurses working at the NICU, also attend the meeting because questions regarding the nursing care could be raised and we need to be there to defend and explain. Claims about nursing care misconduct, like, for example, not giving the prescribed medication or revision of an order or misdiagnosis, could be raised so we will be there to explain. Death audit is a ‘fight’.* (Nurse)

### Fears and expectations of personal punishment and litigation

Even with the experienced discomfort and direct naming, many saw audits as necessary. As an internal system of accountability, audits could ensure that grave mistakes and negligent medical management would be identified and addressed. Some participants also reflected on the need for audits because they did not have any other platform for quality assurance. Participants reported that those who were identified as being responsible for a case got an oral warning directly at the audit meeting in the presence of the other meeting participants. Many also talked about how they feared consequences like having to repeat a year of residency or of losing their job or position at the hospital. None of our informants had, however, experienced this themselves.

The perinatal audit did not function as a system of formal litigation. However, in conversation about mistakes in medical practice, loss of patients, or mismanagement, many participants spontaneously brought up their fears of legal prosecution. Misunderstandings among unqualified personnel or other stakeholders who attended the audits could lead to false rumors that could reach the next-of-kin of deceased neonates and in turn wrongfully expose clinicians to legal action.

*Bad information, sometimes, you know, some people they change the issue, they don’t exactly tell what you told them. They change it and they give it a different meaning and then they spread it to the extent where it affects your life. [...] Fortunately, [this family] was educated and they understand things very well and they just accepted the death. But sometimes, you know, some people may just kill you! They are angry, and they come with a pistol, and they can kill you. Or with a gun… Yes, they think like that, you know. So that’s how, if there is misinformation going on, and it reaches their ear, and then immediately they become emotional and “Oh, my daughter or my wife or my …” is dead because of this, and they start to accuse you. If they are wise enough not to kill you, they may take you to court.* (Senior doctor)

### An exercise in futility

Many participants did not experience that auditing led to improvement in the care provided in their unit. This was a great frustration and restrained their motivation to participate in the audit process. Some participants described this as “the loop not being closed”, referring to examples where mistakes and areas of improvement were identified, but the means for improvement were not made available.

*If it could make one ounce of change I don’t think we would be frustrated, I guess. A little bit of change, a little step in the right direction, if that could be made, perinatal audits would make sense. Right now it is more like we found the problems, we have had them for the past year and a half, 3 years, 4 years, and they still keep happening. We have identified the problem and we don’t have the solution yet, so what is the point?* (Junior doctor)

Test strips for glucometers, appropriate antibiotics for treatment of neonatal sepsis, or simply beds in the labor ward were mentioned as examples of out-of-reach measures for improvement. Furthermore, not being able to apply what is considered up-to-date in the medical literature to their own clinical reality entailed frustration for the involved clinicians. Since the causes of perinatal death and interventions to prevent them were already known, auditing felt like a futile endeavor. In other words, auditing did not provide the means for putting knowledge of causes of perinatal death into preventive action.

*Because I personally always wish for our audits to be actually something that an audit is, I mean, no naming, no finger pointing, no blaming, no shaming. But rather taking up the inputs, the comments, the constructive ideas and putting them on the ground and actually making a difference and having some kind of mechanism to monitor whether the interventions are set in place and if they are working, and then comparing the difference before and after. But I don’t see us actually doing that.* (Senior doctor)

Others mentioned poor roads to hospitals, late referrals, an overwhelming number of patients, and other infrastructural features as examples of improvement points that are out of their control or reach.

*At times, you know, the babies may be neglected; it is not due to the negligence of the physicians, but due to the burden of the patients. So many patients to be seen by a single resident. That is not fair.* (Junior doctor)

### Exceeding sustainable effort and covering your tracks – coping with the burden of death audits

Junior participants disclosed that – out of fear of sanctions from senior doctors and in order to avoid blame from perinatal audits – deaths were sometimes falsely documented in patient charts or pages from patient charts were sometimes deliberately misplaced.

*Academically they will punish us, they will make us repeat the year and sometimes even they will fire us. Fearing the consequences, especially if there is a great mistake in the process that we detect ourselves, we either hide that process or the death. Most of the time we will hide the process, the mistake we made, we report it like it’s a false report. Otherwise, most of the residents do these things.* (Junior doctor)

Providing healthcare at maximal capacity was for many participants an instant reaction and a coping mechanism. All of our informants worked in public hospitals where the numbers of patients were large, and they were often understaffed, leading to little time being available for each patient. They were often unable to ensure sufficient diagnostics and treatments for their patients. Thus, experiences with neonatal loss often led them to be overly cautious, and some reported that they were overtreating patients.

*After a death, when I am going to another mother to care for her, I will overdiagnose. Maybe we will take a fetal heart rate of 120 as normal, but after a loss it is borderline, yeah? So I will intervene as if it is tachycardia because I do not want to have another loss in front of me.* (Junior doctor)

Especially younger doctors found themselves investing increasing amounts of time and energy in academic endeavors and skill improvement outside of working hours, striving to improve their personal practice to avoid further perinatal losses. The discomfort and perceived threat of audit was one underlying aspect of this mechanism.

### Avoidance and absence: clinging to the good, shielding from the bad

When asked about strategies for coping with the burden of the audits, participants also brought up their personal approaches to dealing with perinatal loss and continuous exposure to challenging working conditions. Many described coping through temporarily avoiding complex medical cases or distancing themselves from the hospital environment altogether. One participant, after experiencing a particularly traumatic loss of a mother after a delayed cesarean section, expressed her stress reaction and method of coping as follows:

*I usually disappear from my workplace for a few days, like three days. I cannot talk to people, that is how I shut myself down. Then, I slowly get back into the practice, that is how I do that. So, I literally shy away from the ward. I literally avoid those kinds of cases, unless I am called on, you know? Unless I am called.* (Senior doctor)

Some participants talked about considering leaving their profession or switching or adjusting their jobs to end up in a less stressful environment. Some senior professionals mentioned considering this in their early career, while younger doctors considered it a realistic opportunity, for instance, crossing over to public health, epidemiology, research, pharmacology, or other related ways of using their medical education. Among midwives and nursing staff, this was not as widespread, and some reflected that their options as nurses in the job market were limited.

Most deliveries end well. Knowing that their efforts as clinicians pay off and seeking out and remembering the unproblematic cases and successful deliveries were for many motivating and pushing them to pursue higher quality of care for newborns and laboring mothers. As one participant said:

*The other motivation that I am getting is, as we are facing loss, we are also facing live deliveries, happy mothers, and happy families because of what we are doing. So that will compensate, you know.* (Junior doctor)

Several informants talked about their duty to care for the poor and explained why they continued working under the challenging conditions in public hospitals. One junior doctor spoke about motivation through caring for the worst-off patients like this:

*What keeps me going now, I guess, is I know that the person in front of me wouldn’t be here seeking my help if she had other options. If she could afford private care, you know, where there are two laboring mothers, potentially even none sometimes, and she would be the only person in that hospital and she would be treated like a queen, she would do it. I know that that person in front of me had no choice. Had no other choice than to endure at least 15 other [laboring mothers] on any given night.* (Junior doctor)

## Discussion

Our informants in Ethiopian public hospitals encounter a variety of challenges related to perinatal death, and auditing and inviting them to share their experiences provided a detailed and nuanced picture of what is at stake. The study participants experienced the audit meetings as a burden, and fear of blame and punishment was associated with perinatal death and reviews. For some, covering up information, false documentation, avoiding difficult cases, or absence from work were coping mechanisms. These experiences challenge the purpose of the MPDSR system and could put both patient and clinician safety at risk. Few other studies on the topic are available to date, and generally, the focus has been on maternal death reporting practices [14, 16, 17]. Our study has sought to focus on the perinatal aspect of MPDSR, as perinatal deaths are perceived differently than maternal deaths [39]. Below we discuss our findings in more detail and offer a comparative understanding of our findings in light of existing knowledge and previous studies.

### Blame for “preventable” deaths

Half of all stillbirths globally are considered preventable, and even highly preventable given high-quality care and early risk identification [40, 41]. The WHO’s MPDSR guidelines discuss preventability using the term “modifiable factors” and exemplifies “failure to provide bag and mask in the delivery room” as a modifiable factor [12]. The issue of preventability was important for our study participants, and they also discussed who was responsible for addressing modifiable factors. A frequent example from their practice was the failure to provide appropriate antibiotics on account of their nationwide unavailability. Although perinatal deaths selected for audit reviews are preventable according to normal criteria, our study shows the mismatch between what is *theoretically preventable* and what is *preventable in practice* in the hands of healthcare providers. This discord points to an important unaddressed gap in the discussion on death audit dynamics. When infrastructural limitations in the healthcare system are removed, a focus on individual clinician’s improvement or fault means that these are the only modifiable factors left to identify and discuss in the audit setting.

In our study, causes of and responsibility for deaths were examined with focus on clinicians. As reported in previous studies, few facilities were able to practice a stringent no-blame policy in audit meetings, and naming is viewed by some as a necessary platform to punish responsible clinicians [14, 16]. An implementation study from Tanzania found that even in instances where the auditing environment was declared safe and without blame, junior clinicians were afraid to speak up when mismanagement had taken place [14]. Audits are not detached from the political realities in which they are conducted. Maternal and perinatal deaths are sensitive issues, and the implementation of MPDSR systems interacts with existing health system power dynamics and hierarchies [42]. The impact of complex hierarchies and power dynamics in healthcare staff meetings should be further explored because the fear of being “put on the spot” might not be related only to death reviews.

A recurring issue from our findings is the absence of systems for appropriately dealing with individual malpractice or health system-level failures, as previously shown [43]. In the patient safety literature, incidents are traditionally viewed as due to either individual errors or system-level errors, the latter often exemplified by service inaccessibility, unavailable resources, and adverse working hours [44, 45]. Our study participants feared personal blame for perinatal deaths, and fear of blame and litigation are well-known barriers to reliable reporting of incidents in different areas of healthcare [15, 45]. None of the participants had personal experience with it, but *the fear of* legal prosecution alone was a powerful and problematic barrier.

### Coping with death and death reviews

Experiencing perinatal deaths on a frequent basis is in itself demanding, and perinatal death audits can be regarded as a double burden for the involved clinicians. Several methods of coping with perinatal death were relevant for our informants, such as avoiding work, avoiding difficult cases or seeking out good cases, overworking, and making false documentation. Many studies have explored healthcare professionals’ experiences with perinatal loss and have reported various coping styles, including increased work focus and effort or debriefing with colleagues or other clinicians [46]. Knowledge about coping in the face of perinatal deaths in the African setting is limited, thus indicating the need for further qualitative exploration and clinician support [47].

In Rushton’s work on moral resilience and ‘fight/flight/freeze’ responses to stress, ‘fight’ is described as strategies to take control over the source of distress, ‘flight’ as disengaging from the distressful situation, and ‘freeze’ as numbing out relevant emotions or avoiding the source of distress [48]. Seeing the coping strategies described by our participants through this understanding could be a useful approach. Increasing work effort to maximal capacity equals a fight response. ‘Fight’ also includes overtreating patients and continuously striving for skill improvement outside working hours. Flight responses, like downplaying the severity or importance of perinatal death or seeking out the good/easy cases, were also described. Avoiding complicated cases, switching or leaving the clinical professions, and false documentation of perinatal death could all be understood as forms of freeze reactions.

As long as blame remains central, providing maternal and perinatal healthcare and conducting perinatal death audits exposes healthcare workers to moral distress as a continuous situation. While healthcare professionals absorb the consequences of structural limitations of the system and look for improvement actions within themselves, the source of moral injury resides in the system or environment in which they work [49]. The capacity for cultivating moral resilience is, in our opinion, restricted by a wounded system. Without the opportunity to heal, fostering moral resilience is neither easy nor a priority for healthcare providers working in a frail system. We argue that more resources need to be allocated to training in tackling suboptimal working conditions and ethical dilemmas are necessary to foster moral resilience and prevent professional fatigue among Ethiopian healthcare professionals.

### Balancing the benefits and burdens of death audits

While the MPDSR is constructed to highlight improvable aspects locally as well as at the system level, our participants experienced that areas in need of improvement did not receive the warranted attention, even if they were audited. Other studies on sub-Saharan MPDSR implementation have found that only some facilities are able to suggest action plans as a result of death reviews [16, 17]. When actions for improvement are suggested few of them are specific or feasible enough to represent a real response in the MPDSR cycle. A recent study from Uganda that analyzed perinatal death review/auditing as an intervention found a statistically significant reduction in neonatal death rates when isolated from perinatal death rates but no significant effect of auditing on outcomes of fatality from prematurity, hypoxia, infection, or stillbirth [50]. Similarly, a Cochrane review on maternal death audits concluded that maternal death review alone is not sufficient to reduce maternal mortality [51]. The authors concluded that perinatal death reviews likely have the greatest potential to reduce death rates in countries with already low perinatal mortality and high access to resources.

Our study did not evaluate audit as an intervention, and we cannot draw any conclusions about its effect on perinatal mortality rates at our study sites. In accordance with our findings, other studies have found that a lack of follow-up actions resulting from review meetings reduces the motivation to conduct reviews among healthcare providers and leads to repetition of the same issues without the appropriate means of improvement [14, 15, 18]. The need for clinical guidelines is particularly pronounced in settings where resource rationing dilemmas are common [52, 53]. For perinatal death audits to generate tangible responses, action plans and guidelines need to be contextualized and need to take into account both resource availability and other structural conditions. To further alleviate Ethiopian clinicians of this double burden, the guidelines should explicitly clarify areas of responsibility. These clinicians are the main actors and important stakeholders in perinatal health, and they should be consulted in the synthesis of context-sensitive guidelines and action plans.

### Strengths and limitations of the study

The main limitations of our study are related to challenges with fieldwork. Recruitment of more participants could have enabled even richer data material toward thematic saturation. We were not able to include qualitative observations in the field, which we believe would have further strengthened the study and the methodological triangulation. In addition, we recognize that maternal and perinatal deaths are sensitive subjects and that some participants may hesitate to share freely about their experiences. However, we found that having one interviewing author with status as an outsider in the Ethiopian culture and context was a strength. This allowed for easier confidence and disclosure of sensitive information. The other interviewing author brought familiarity and knowledge about local culture and customs, thus strengthening our analytical ability and contextual understanding.

## Conclusion

The intention of perinatal death audits through the MPDSR system is to learn from mistakes and undesirable circumstances, recommend follow-up actions, and implement actions with the end goal being to improve the quality of healthcare. We have examined practices of perinatal death auditing and healthcare professionals’ perceptions and responses at the facility level in Ethiopia. With the current practices and barriers, experiencing perinatal death and death auditing constitutes a double burden for the involved clinicians. Preventability and its status as a universal concept are widely discussed and are closely linked to clinicians’ experiences of blame and worries about legal action. We asked our participants for ways of coping with this double burden, and their strategies were constructive to varying degrees, indicating that there are limits to what the individual Ethiopian clinician should tackle alone. Cultivation of moral resilience and strengthened handling of ethical dilemmas require proper training and the allocation of more resources for training. Context-specific guidelines are necessary to relieve healthcare professionals from added burdens in their clinical practice. Guidelines that are sensitive to resource-related and system-related challenges may support healthcare professionals in constructing feasible action plans for tangible responses and thus for improvements in perinatal health.  

## Data Availability

The datasets generated and analyzed during the current study are not publicly available due to protection of study participants identity, but request to the corresponding author may be considered upon reasonable request.
